# Association between *NAT2*, *CYP1A1*, and *CYP1A2* genotypes, heterocyclic aromatic amines, and prostate cancer risk: a case control study in Japan

**DOI:** 10.1186/s12199-017-0681-0

**Published:** 2017-10-24

**Authors:** Masahide Koda, Motoki Iwasaki, Yuko Yamano, Xi Lu, Takahiko Katoh

**Affiliations:** 1 Department of Public Health, Faculty of Life Sciences, Kumamoto University, 1-1-1 Honjou, Chuo-ku, Kumamoto, 860-8556 Japan; 2 Division of Epidemiology, Center for Public Health Sciences, National Cancer Center, Tokyo, Japan; 3 Department of Hygiene and Preventive Medicine, Showa University School of Medicine, Tokyo, Japan

**Keywords:** Prostate cancer, *NAT2*, *CYP1A1*, *CYP1A2*, Heterocyclic aromatic amines

## Abstract

**Background:**

Heterocyclic aromatic amines (HAAs) may confer prostate cancer risk; however, the evidence is inconclusive and the activity of HAA-metabolizing enzymes is modulated by gene variants. The purpose of our study was to determine whether there was evidence of an association between HAA intake, polymorphisms in *NAT2*, *CYP1A1*, and *CYP1A2* and prostate cancer risk in Japanese men.

**Methods:**

Secondary data analysis of an observational case control study was performed. Among 750 patients with prostate cancer and 870 healthy controls, 351 cases and 351 age-matched controls were enrolled for analysis. HAA intake was estimated using a food frequency questionnaire and genotypes were scored by TaqMan real-time PCR assay. Logistic regression analysis was conducted according to affected/control status.

**Results:**

We found that high HAA intake was significantly associated with an increased risk of prostate cancer (odds ratio (OR), 1.90; 95% confidence interval (95% CI), 1.40–2.59). The increased risk of prostate cancer was observed among individuals with the *NAT2* slow acetylator phenotype (OR, 1.65; 95% CI, 1.04–2.61), *CYP1A1* GA + GG genotype (OR, 1.27; 95% CI, 1.02–1.59), and *CYP1A2* CA + AA genotype (OR, 1.43; 95% CI, 1.03–2.00). In addition, *CYP1A1* GA + GG genotypes were associated with increased cancer risk in low (OR, 2.05; 95% CI, 1.19–3.63), moderate (OR, 1.72; 95% CI, 1.07–2.76), and high (OR, 2.86; 95% CI, 1.83–4.47) HAA intake groups.

**Conclusions:**

Our results suggest that high HAA intake is a risk factor of prostate cancer, and genotypes related to HAA metabolic enzymes can modulate the degree of the risk.

## Background

It has been proposed that high consumption of meat, including red and processed meat as well as fish, confers risk of prostate cancer [1–5]. Heterocyclic aromatic amines (HAAs), which are known mutagens formed in cooked meat and fish at high temperatures, are considered the causative agents for the association between meat intake and prostate cancer risk [2, 6–8].

HAAs can metabolically result in carcinogenesis through the formation of HAA-DNA adducts [9–11], which lead to tumor formation by conferring mutations in genes that control cell proliferation [12]. In addition, it was shown that human prostate cells metabolize HAAs to a carcinogenic state by forming HAA-DNA adducts after exposure to HAAs in vivo [8, 13–16].

Several studies have investigated whether there is an association between dietary HAA intake and prostate cancer risk; however, results were suggestive yet inconclusive [1, 3, 17, 18]. This disparity reflects two main reasons. First, it is difficult to assess dietary HAA intake, the composition of which varies according to the cooking method and meat type [19, 20]. In Japan, several studies investigated prostate cancer risk, using only meat and fish intake [21, 22]; however, no prior study has investigated the risk by estimating the dietary HAA intake. Typically, Japanese elderly people intake more fish than meat, and they prefer chopping and stir-frying their meat to grilling [23, 24]. Foods that contribute to HAA intake differ by country [25]; therefore, dietary assessment tools for HAA intake should be tailored specifically to the population being studied.

The second reason is the difficulty in assessing a study population in terms of metabolic variation determined by genetic heterogeneity. Similar to other environmental chemical carcinogens, it is necessary for HAAs to be metabolically activated by host enzymes to acquire genetic toxicity. Phase I enzymes such as cytochrome P450 (CYP) enable HAAs to metabolically activate and, thus, form genotoxic electrophilic intermediates [26]. This transition enables phase II enzymes, including *N*-acetyltransferase 1 (*NAT1*) and *N*-acetyltransferase 2 (*NAT2*), to detoxify part of the activated metabolites by performing the tasks of N-acetylation and O-acetylation [27]. It is presumed that the relative activities of these metabolizing enzymes, which are mostly genetically determined, have a critical role in HAA-mediated prostate cancer development.

In the phase I cytochrome P450 family, *CYP1A1* and *CYP1A2* are highly active in the liver and play a major role in the metabolic activation of HAAs, and each enzyme activity has been considered to be linked to genetic variations [18, 28, 29]. In phase II enzymes, previous studies suggested that the genetic polymorphisms in *NAT1* and/or *NAT2* may modify prostate cancer risk related to exposure to HAA carcinogens [30]. The frequencies of *NAT1* and *NAT2* genotype varies according to racial and ethnic background, and the frequency difference may be a factor in cancer incidence [31]. Although *NAT1* is expressed in the prostate, the relationship between the *NAT1* genotype and phenotype in a Japanese population remains unclear [32]. On the other hand, *NAT2* is expressed predominantly in the liver, and the relationship between genetic variants of *NAT2* and the acetylator phenotype in Japanese individuals is clear [31, 33–35]. It is considered that the *NAT2* slow acetylator phenotype would increase the prostate cancer risk because this phenotype would have reduced hepatic N-acetylation for detoxification of HAA carcinogens, thus increasing the chance of hepatic N-hydroxylation for activation [36].

Therefore, in this study, we used a validated assessment food frequency questionnaire (FFQ) to assess dietary HAAs in Japanese cultural contexts [25, 37, 38] and investigated the phenotypes and genotypes of *NAT2*, *CYP1A1*, and *CYP1A2* in our subjects. To the best of our knowledge, no prior reports have investigated the relationship between *NAT2*, *CYP1A1*, and *CYP1A2* polymorphisms and HAAs as risk factors for prostate cancer in a Japanese population. The purpose of this study was to determine the impact of HAA intake and genetic polymorphisms in *NAT2*, *CYP1A1*, and *CYP1A2* on prostate cancer in Japanese men.

## Methods

### Design

This study was a secondary data analysis of an observational case control study. Written informed consent was obtained from all subjects by the return of the questionnaires. The study protocol received ethics approval by the institutional review board of Faculty of Life Sciences, Kumamoto University (Kumamoto, Japan), on June 10, 2016 (approval number 209). The first and last authors take all responsibility for the integrity of the data and the accuracy of the data analysis.

### Subjects

We identified 750 eligible male patients between 40 to 90 years of age who were diagnosed with histologically confirmed prostate cancer at the Jikei University School of Medicine Hospital (Tokyo, Japan) from August 2004 to December 2006. We also identified 870 men as prospective controls in the same age range who (i) underwent comprehensive medical examination for health screening at the Mitsui Memorial Hospital (Tokyo, Japan) during the same survey period, (ii) exhibited no evidence of prostate cancer as determined by blood test (PSA < 4 mg/dL), and (iii) provided consent for participation. Because age is a known risk factor for prostate cancer, we selected age-adjusted control subjects according to the average age of our affected group and identified 351 cases and 351 controls for analysis.

### Exposure assessment

Subjects completed a self-administered questionnaire regarding general characteristics: age, sex, height, weight, occupation, smoking and drinking habits, dietary habits, personal medical history, and family history including prostate cancer. Food and beverage consumption was assessed with a food frequency questionnaire (FFQ) [23] that consists of 138 food and beverage items, which asks to report the usual consumption of the listed foods during the past year. Frequency response choices for food items consisted of nine options: less than once per month, 1–3 times per month, 1–2 times per week, 3–4 times per week, 5–6 times per week, once per day, 2–3 times per day, 4–6 times per day, and 7 or more times per day. Further, standard portion sizes were selected for each food item with three choices: medium (standard), small (50% smaller), and large (50% larger).

HAA consumption is a different parameter in meat and fish consumption. For fish, HAA intake was estimated from 19 kinds of items: salted fish, semi-dried split fish, salmon, horse mackerel or sardines, Pacific saury or mackerel, and eel from six groups. In addition, participants were asked about grilled skin consumption using five quantity categories: almost none, one-third, half, two-thirds, and almost all. HAA intake from fish consumption was then estimated based on the proportion of grilled to total fish consumption, the rate of grilled skin consumption, the ratio of skin-to-flesh, and data on HAA content in the skin and flesh.

Similarly, HAA intake from meat was estimated according to 7 of 18 meat items: pan-fired and grilled beef, stir-fried pork and pork liver, grilled chicken and chicken liver, and bacon. Participants reported their preferred grilled level of pan-fried and grilled beef among five categories: very well-done, well-done, medium, medium rare, and rare. HAA intake consumption was then estimated based on this information and data on HAA content, which were validated measurements of 2-amino-1-methyl-6-phenylimidazo[4,5-b]pyridine (PhIP) levels in the hair from a prior study [25, 37, 38].

### Genotyping

Blood samples were obtained from all participants and collected during the protocol period before a procedure was performed. Buffy coats were preserved at − 80 °C soon after blood sampling until analysis. Pre-validated allelic discrimination TaqMan real-time PCR (Applied Biosystems, Foster City, CA, USA) was used for genotyping of single nucleotide polymorphisms (SNPs). The reaction solution (9 μL) was aliquoted into each well of a 48-well reaction plate, and 1 μL of DNA or water control was added to each tube.

*NAT2* slow acetylator alleles were reported as: NAT2*5 (rs1801280: T > C), NAT2*6 (rs1799930: G > A), and NAT2*7 (rs1799931: G > A). Although NAT2*13 (rs1208: A > G) was previously considered among slow acetylator alleles, recent studies have indicated that alleles of NAT2*4 and NAT2*13 dominantly determine the fast acetylator phenotype [31–35]. Slow acetylators were classified if individuals have at least two variant alleles, and intermediate acetylators were categorized as those individuals having one slow acetylator allele from these loci. Genotyping of rs1048943 (A > G) at *CYP1A1* and rs762551 (A > G) at *CYP1A2* was also undertaken.

### Statistical analysis

For demographic and clinical data, we used the chi-square test to compare proportions of categorical variables and the Mann–Whitney test to compare the averages of continuous variables between groups. Conditional logistic regression models were used to evaluate the relationship between HAA intake and genotype. Our models were adjusted for variables associated with prostate cancer identified either by the current study or by previous studies [39–41] and included alcohol consumption, smoking status, body mass index (BMI), family history of prostate cancer, and total energy intake. Intake of HAA and other nutrients and foods was adjusted for total energy intake using the residual model [42]. HAA intake was divided into tertile categories and evaluated using the Cochran–Armitage test for trend. To identify associations between HAA intake and polymorphisms of tested genes, we assessed HAA intake based on subgrouping subjects as “rapid/intermediate” or “slow” for *NAT2* as well as by variant alleles, and by those with two wild-type alleles for *CYP1A1* and *CYP1A2*. The threshold for significance was *P* < 0.05. All statistical analyses were conducted using SPSS version 21 (IBM Corp., Armonk, NY, USA).

## Results

The general characteristics of our cohorts are presented in Table 1. This study included 351 patients with prostate cancer with a pathologically confirmed diagnosis and 351 cancer-free controls, aged from 50 to 79 years of age. The average age in both groups was 64.9 years. We observed that a significantly higher rate of family history of prostate cancer was found in cases compared to that in controls, while the rate of alcohol consumption was lower in cases compared to that in controls. Although we found no differences in BMI and total energy intake between the two groups, we found significantly higher rates of the following measures in cases compared to those in controls: total HAA intake; 3-amino-1, 4-dimethyl-5H-pyrido[4, 3-b]indole (Trp-P-1); 2-amino-3-methylimidazo[4, 5-f]quinoline (IQ); 2-amino-3, 4-dimethylimidazo[4, 5-f]quinoline (MeIQ); 2-amino-3, 8-dimethylimidazo[4, 5-f]quinoxaline (MeIQx); and PhIP. We found no statistically significant differences between cohorts for other variables including meat, red meat, processed meat, vegetable, and salt (NaCl) intake as well as the rate of those who prefer their meat well done or ate almost all burnt fish skin.

**Table 1:** Characteristics of cases and controls

Characteristics	Cases	Controls	*P*
Number	351	351	
Age, years; mean (SD)	64.9 (0.35)	64.9 (0.35)	1.00^b^
< 65; *n* (%)	180 (51.3%)	180 (51.3%)	
≥ 65; *n* (%)	171 (48.7%)	171 (48.7%)	1.00^a^
Family history of prostate cancer; *n* (%)	13 (3.7%)	4 (1.1%)	0.027^a^
Alcohol consumption, g/week; mean (SD)	151.6 (192.4)	196.0 (207.2)	< 0.001^b^
Smoking status; *n* (%)			
Never	126 (35.9%)	108 (30.8%)	
Ever	225 (64.1%)	243 (69.2%)	0.15^a^
Current smoker	55 (15.7%)	67 (19.1%)	0.23^a^
Ex-smoker	170 (48.4%)	176 (50.1%)	0.65^a^
BMI, kg/m^2^; mean (SD)	23.8 (0.14)	23.8 (0.13)	1.00^b^
Total HAA intake, ng/day; mean (SD)	46.5 (1.6)	38.2 (1.7)	< 0.001^b^
Trp-P-1 intake	3.9 (0.23)	3.1 (0.19)	< 0.001^b^
IQ intake	0.32 (0.021)	0.27 (0.018)	0.012^b^
MeIQ intake	5.9 (0.21)	4.8 (0.17)	< 0.001^b^
MeIQx intake	6.4 (0.21)	5.2 (0.20)	< 0.001^b^
4,8-DiMelQx intake	0.41 (0.34)	0.36 (0.33)	0.10^b^
7,8-DiMelQx intake	1.6 (0.14)	1.4 (0.14)	0.06^b^
PhIP intake	28.2 (0.98)	23.3 (0.93)	< 0.001 ^b^
Total energy intake, kcal/day; mean (SD)	1869.2 (567.8)	1789.3 (519.9)	0.30^b^
Meat intake, g/day; mean (SD)	59.9 (41.0)	50.2 (36.1)	0.84^b^
Red meat intake, g/day; mean (SD)	47.3 (33.8)	37.6 (31.0)	0.87^b^
Processed meat intake, g/day; mean (SD)	7.1 (7.9)	6.3 (8.6)	0.42^b^
Fish intake, g/day; mean (SD)	84.2 (58.3)	77.4 (62.8)	0.51^b^
Vegetable intake, g/day; mean (SD)	88.7 (68.4)	82.7 (73.2)	0.24^b^
NaCl intake, g/day; mean (SD)	10.1 (4.5)	9.2 (4.1)	0.79^b^
Participants with preference on meat cooked level as well done or very well done (%)	33 (50.7%)	26(44%)	0.40^a^
Participants with preference on eating almost all of grilled fish skin (%)	71 (50.4%)	70 (49.6%)	0.99^a^

*BMI* body mass index; *SD* standard deviation; *HAA* heterocyclic aromatic amine; *NaCl* sodium chloride; *Trp-P-1* 3-Amino-1, 4-dimethyl-5H-pyrido[4, 3-b]indole; *IQ* 2-Amino-3-methylimidazo[4,5-f]quinoline; *MeIQ* 2-Amino-3,4-dimethylimidazo[4,5-f]quinoline; *MeIQx* 2-Amino-3,8-dimethylimidazo[4,5-f]quinoxaline; *4,8-DiMelQx* 2-Amino-3, 4, 8-trimethylimidazo[4, 5-f]quinoxaline; *7,8-DiMelQx* 2-Amino-3, 7, 8-trimethylimidazo[4, 5-f]quinoxaline; *PhIP* 2-Amino-1-methyl-6-phenylimidazo[4,5-b]pyridine

^a^Chi-square test

^b^Mann-Whitney test

As shown in Table 2, the distribution of genotyped SNPs was NAT2*5 (rs1801280), NAT2*6 (rs1799930), NAT2*7 (rs1799931), NAT2*13 (rs1208), *CYP1A1* (rs1048943), and *CYP1A2* (rs762551). Genotype frequencies of each SNP were consistent with Hardy–Weinberg equilibrium among controls.

**Table 2:** SNPs and their allele frequencies in *NAT2*, *CYP1A1*, and *CYP1A2*

	SNP rs #	Amino acid change	Major/minor allele	Major allele frequency in control group
*NAT2*				
NAT2*5	rs1801280	Ile114Thr	T/C	0.986
NAT2*6	rs1799930	Arg197Gln	G/A	0.96
NAT2*7	rs1799931	Gly286Glu	G/A	0.641
NAT2*13	rs1208	Lys268Arg	A/G	0.826
*CYP1A1*	rs1048943	Ile462Val	A/G	0.661
*CYP1A2*	rs762551	5′-UTR	A/C	0.379

*UTR* untranslated region, *SNP* single nucleotide polymorphism

Multivariate odds ratios (ORs) for HAA intake were analyzed, and several HAA-related measures with high intake were significantly associated with an increased risk of prostate cancer compared to controls (Table 3): total HAA (OR, 1.90; 95% confidence interval (95% CI), 1.40–2.59), Trp-P-1 (OR, 1.92; 95% CI, 1.42–2.61), MeIQ (OR, 1.87; 95% CI, 1.38–2.55), MeIQx (OR, 2.25; 95% CI, 1.65–3.06), and PhIP (OR, 1.84; 95% CI, 1.35–2.50).

**Table 3:** Association between HAA intake and prostate cancer

	Mean (SD)	Cases	Controls	OR (95% CI)^a^
Total HAAs (ng/day)				
Low tertile	17.5 (7.1)	100	131	1.00
Moderate tertile	35.6 (5.3)	108	124	1.14 (0.83–1.58)
High tertile	72.9 (29.2)	143	96	1.90 (1.40–2.59)
Trend P^b^				< 0.001
Total Trp-P-1 (ng/day)				
Low tertile	0.44 (0.55)	104	129	1.00
Moderate tertile	2.9 (0.55)	104	128	1.05 (0.76–1.45)
High tertile	7.2 (5.0)	143	94	1.92 (1.42–2.61)
Trend P				< 0.001
Total MeIQ (ng/day)				
Low tertile	2.3 (0.93)	100	131	1.00
Moderate tertile	4.5 (0.57)	109	123	1.18 (0.85–1.63)
High tertile	9.0 (3.7)	142	97	1.87 (1.38–2.55)
Trend P				< 0.001
Total MeIQx (ng/day)				
Low tertile	2.4 (0.98)	99	132	1.00
Moderate tertile	4.9 (0.71)	102	130	1.05 (0.76–1.46)
High tertile	9.9 (3.9)	150	89	2.25 (1.65–3.06)
Trend P				< 0.001
Total PhIP (ng/day)				
Low tertile	10.5 (4.2)	102	129	1.00
Moderate tertile	21.5 (3.2)	106	126	1.06 (0.77–1.48)
High tertile	44.7 (18.3)	143	96	1.84 (1.35–2.50)
Trend P				< 0.001

*BMI* body mass index; *OR* oratio; *95% CI* 95% confidence interval; *HAA* heterocyclic aromatic amine; *Trp-P-1* 3-Amino-1, 4-dimethyl-5H-pyrido[4, 3-b]indole; *MeIQ* 2-Amino-3,4-dimethylimidazo[4,5-f]quinoline; *MeIQx* 2-Amino-3,8-dimethylimidazo[4,5-f]quinoxaline; *PhIP* 2-Amino-1-methyl-6-phenylimidazo[4,5-b]pyridine

^a^ORs (95% CI) adjusted by conditional logistic regression for alcohol intake, smoking status, BMI, family history of prostate cancer, and total energy intake

^b^ *P* value for Cochran–Armitage test for trend

Multivariate ORs for the *NAT2*-imputed phenotype and the *CYP1A1* and *CYP1A2* genotypes are shown in Table 4. We found that the factors associated with a significantly increased risk of prostate cancer were the *NAT2* slow acetylator phenotype (OR, 1.65; 95% CI, 1.04–2.61), the *CYP1A1* GG genotype (OR, 1.76; 95% CI, 1.08–2.87), the *CYP1A1* GA + GG genotype (OR, 1.27; 95% CI, 1.02–1.59), the *CYP1A2* CA genotype (OR, 1.43; 95% CI, 1.01–2.03), the *CYP1A2* AA genotype (OR, 1.44; 95% CI, 1.00–2.06), and the *CYP1A2* CA + AA genotype (OR, 1.43; 95% CI, 1.03–2.00).

**Table 4:** Association between *NAT2*-imputed phenotype, *CYP1A1* and *CYP1A2* genotype, and prostate cancer

	Cases	Controls	*P*	OR (95% CI)^a^
*NAT2*-imputed phenotype				
Rapid acetylator	206	212		1.00
Intermediate acetylator	118	121	0.69	1.05 (0.83–1.32)
Slow acetylator	27	18	0.034	1.65 (1.04–2.61)
*CYP1A1*				
AA	207	232		1.00
GA	119	105	0.12	1.20 (0.95–1.52)
GG	25	14	0.023	1.76 (1.08–2.87)
GA + GG	144	119	0.034	1.27 (1.02–1.59)
*CYP1A2*				
CC	34	47		1.00
CA	177	171	0.045	1.43 (1.01–2.03)
AA	140	133	0.048	1.44 (1.00–2.06)
CA + AA	317	304	0.035	1.43 (1.03–2.00)

*BMI* body mass index, *OR* odds ratio, *CI* confidence interval

^a^OR (95% CI) adjusted by conditional logistic regression for alcohol intake, smoking status, BMI, family history of prostate cancer, and total energy intake

Analyses of combinations between HAA intake and *NAT2*-imputed phenotype and *CYP1A1* and *CYP1A2* genotypes are shown in Table 5. High HAA intake was associated with an increased risk of prostate cancer among each genotype and NAT-imputed phenotype, with the exception of the *CYP1A2* CC genotype (OR, 2.65; 95% CI, 0.97–7.29; *P* for interaction, 0.003). In addition, among high HAA intake individuals, the risk was indicated by the phenotypes or genotypes, which were suggested from the result of Table 4. *NAT2* slow acetylator phenotype, *CYP1A1* GA + GG, and *CYP1A2* CA + AA had higher OR than other paired phenotypes or genotypes as follows: the *NAT2* slow acetylator phenotype (OR, 5.29; 95% CI, 2.37–11.80; *P* for interaction, 0.004) was higher than the rapid or intermediate acetylator phenotype (OR, 1.85; 95% CI, 1.34–2.56); the *CYP1A1* GA + GG genotype (OR, 2.86; 95% CI, 1.83–4.47) was higher than the AA genotype (OR, 2.41; 95% CI, 1.61–3.63); and the *CYP1A2* CA + AA genotype (OR, 4.01; 95% CI, 1.60–10.05) was higher than the CC genotype (OR, 2.65; 95% CI 0.97–7.29) even though the CC genotype OR is not statistically significant. Among individuals from the *CYP1A1* GA + GG genotype group (*P* for interaction, < 0.001), we found there was an association between increased risk of prostate cancer and all three HAA intake groups specifically low (OR, 2.05; 95% CI, 1.19–3.63), intermediate (OR, 1.72; 95% CI, 1.07–2.76), and high (OR, 2.86; 95% CI, 1.83–4.47).

**Table 5:** Prostate cancer risk and HAA intake stratified by *NAT2*-imputed phenotype and *CYP1A1* and *CYP1A2* genotype

	HAA intake			
	Low	Moderate	High	*P*_interaction_
*NAT2*-imputed phenotype				
Rapid or intermediate acetylator				
Cases/Controls	92/124	100/116	132/93	
OR (95% CI)^a^	1.00	1.17 (0.84–1.65)	1.85 (1.34–2.56)	
Slow acetylator				
Cases/Controls	8/7	8/8	11/3	
OR (95% CI)	1.43 (0.50–4.12)	1.21 (0.56–2.60)	5.29 (2.37–11.80)	0.004
*CYP1A1*				
AA				
Cases/controls	51/87	68/82	88/63	
OR (95% CI)	1.00	1.46 (0.95–2.24)	2.41 (1.61–3.63)	
GA + GG				
Cases/controls	49/44	40/42	55/33	
OR (95% CI)	2.05 (1.19–3.63)	1.72 (1.07–2.76)	2.86 (1.83–4.47)	< 0.001
*CYP1A2*				
CC				
Cases/controls	7/17	14/17	13/13	
OR (95% CI)	1.00	2.05 (0.73–5.77)	2.65 (0.97–7.29)	
CA + AA				
Cases/controls	93/114	94/107	130/83	
OR (95% CI)	2.17 (0.85–5.59)	2.34 (0.93–5.92)	4.01 (1.60–10.05)	0.003

*BMI* body mass index, *OR* odds ratio, *CI* confidence interval, *HAA* heterocyclic aromatic amine

^a^OR (95% CI) adjusted by conditional logistic regression for alcohol intake, smoking status, BMI, family history of prostate cancer, and total energy intake

## Discussion

This is the first national study which investigates the association between dietary HAA intake by using FFQ for estimate the amount of HAA, HAA-metabolic polymorphisms, and prostate cancer risk in Japanese people. We found that a high HAA intake was significantly associated with an increased risk of prostate cancer (Table 3), and the *NAT2* slow acetylator phenotype, the *CYP1A1* GG and GA + GG genotypes, and the *CYP1A2* CA, AA, and CA + AA genotypes were associated with an increased prostate cancer risk in our affected cohort with compared with the controls (Table 4). In addition, the *CYP1A1* GA + GG genotype was associated with prostate cancer in low, moderate, and high HAA intake groups (Tables 4 and 5). These results support our hypothesis that high HAA intake is a risk factor of prostate cancer, and the degree of risk is influenced by polymorphisms in genes encoding HAA metabolic enzymes.

Previous studies have shown confounding results on whether there is an association between HAA intake and prostate cancer risk [1, 3, 17, 18]. In Japanese, several studies specifically investigated the risk of only meat and fish intake to prostate cancer [21, 22], but no prior study investigated the association between dietary HAAs and prostate cancer risk in a Japanese population. The main reason for the absence of such a study is because of the difficulty in assessing dietary HAA intake. The HAA composition of cooked meat and fish varies depending on the cooking method and meat type [19, 20]. In addition, typical elderly Japanese intake more fish than meat, and they tend to prefer chopping and stir-frying meat than grilling [23, 24]. Furthermore, foods that contribute to HAA intake differ by country [25] and, therefore, dietary assessment tools for HAA intake should be tailored specifically for the population being studied. In this study, we used a validated assessment FFQ to assess dietary HAAs in our Japanese cohorts [25, 37, 38], and we found that high HAA intake was associated with an increased risk of prostate cancer as follows: total HAA intake (OR, 1.90; 95% CI, 1.40–2.59), Trp-P-1 (OR, 1.92; 95% CI, 1.42–2.61), MeIQ (OR, 1.87; 95% CI, 1.38–2.55), MeIQx (OR, 2.25; 95% CI, 1.65–3.06), and PhIP (OR, 1.84; 95% CI, 1.35–2.50) (Table 3). In previous studies, which were not Japanese cohort research, no data or significant associations in prostate cancer were observed for dietary Trp-P-1, MeIQ, or MeIQx [3, 4, 43, 44]. A statistically significant association between PhIP and prostate cancer risk was observed in one study [44]; however, null or opposed associations were reported in another study [3, 4, 45]. Thus, the present study is the first to report an association between dietary HAA intake and prostate cancer in a Japanese cohort, and it is epidemiologically plausible that high HAA intake is a risk factor of prostate cancer in Japan.

Regarding HAA metabolism, *NAT2* is a phase II enzyme that detoxifies HAA carcinogens, is expressed predominantly in the liver, and is highly polymorphic in humans [13]. It was observed that the frequency of *NAT2* genotypes varies by population [27]. A previous study showed that the *NAT2* slow acetylator phenotype presents decreased enzymatic activity compared with the activity levels of the rapid or intermediate genotypes [46]. As a result, the *NAT2* slow acetylator phenotype is considered to reduce hepatic N-acetylation (detoxification) and increase the chance of hepatic N-hydroxylation (activation) [31]. One meta-analysis showed that there was no evidence of an association between *NAT2* polymorphisms and prostate cancer in a combined analysis, but there was an association in Asian populations based on racial subgroup analysis [47]. In our study, we found that the *NAT2* slow acetylator phenotype was associated with an increased risk of prostate cancer (OR, 1.65; 95% CI, 1.04–2.61) (Table 4) and that the *NAT2* slow acetylator phenotype (OR, 5.29; 95% CI, 2.37–11.80; *P* for interaction, 0.004) was higher than rapid or intermediate acetylator phenotypes (OR, 1.85; 95% CI, 1.34–2.56) among individuals with high HAA intake (Table 5). We posit that this finding indicates that individuals with the *NAT2* slow acetylator phenotype have reduced detoxification of HAA carcinogens and increased chance of carcinogen activation, compared with other acetylator phenotypes in individuals with prostate cancer. Although *NAT2* is expressed in prostate epithelium and the liver [13], our results suggest that the expression of the *NAT2* phenotype in the liver has greater metabolic influence on HAA carcinogens of prostate cancer than its expression in the prostate.

*CYP1A1* is a member of the phase I cytochrome P450 family, which is highly active in the liver, acting on the metabolic activation of HAAs [28]. Similar to *NAT2*, *CYP1A1* mRNA is also expressed in prostate tissue [48–50]. *CYP1A1* in individuals harboring the mutant G allele (GA or GG genotype) in exon 7, which substitutes valine for isoleucine, has a higher catalytic activity compared to that found in individuals who are homozygous AA [51]. Findings from a previous study suggested that the *CYP1A1* GA + GG genotype confers susceptibility to prostate cancer and that individuals with the *CYP1A1* GG genotype have a significantly increased risk [29, 52]. Further, a meta-analysis study showed using subgroup analysis that the *CYP1A1* GA + GG genotypes were associated with prostate cancer risk in Asians [53]. Similarly, in our study, we found that the *CYP1A1* GA + GG genotypes were associated with prostate cancer risk (OR, 1.27; 95% CI, 1.02–1.59) (Table 4), and this evidence of an association was observed not only in the high HAA intake group (OR, 2.86; 95% CI, 1.83–4.47), but also in even the low (OR, 2.05; 95% CI, 1.19–3.63) and moderate (OR, 1.72; 95% CI, 1.07–2.76) HAA intake groups (Table 5). These findings provide further support that there is a relationship between *CYP1A1* GA + GG genotypes and an enhanced effect on HAA carcinogens in prostate cancer. The second possibility is that this *CYP1A1* polymorphism is an independent risk factor for prostate cancer.

Similar to *CYP1A1*, *CYP1A2* is a phase I metabolizing enzyme that plays a prominent role in the activation of HAA carcinogens [28], and it is also expressed in prostate tissue [49, 50]. *CYP1A2* in individuals with the A allele (CA or AA genotype) has a higher enzymatic activity compared to that found in individuals who are homozygous CC [54]. A previous study showed that the *CYP1A2* CC genotype may be associated with risk of prostate cancer but the finding was not statistically significant [29]. It is unclear how reduced *CYP1A2* activity contributes to an increased risk of prostate cancer. Nevertheless, in contrast to past findings, we found that the *CYP1A2* AA, CA, and CA + AA genotypes were associated with an increased risk of prostate cancer (Table 4) and that the *CYP1A2* CA + AA genotype (OR, 4.01; 95% CI, 1.60–10.05; *P* for interaction, 0.003) was higher than the CC genotype (OR, 2.65; 95% CI, 0.97–7.29) among individuals with high HAA intake, even though the CC genotype OR is not statistically significant, we still can find the trend (Table 5). These findings support that *CYP1A2*-induced higher activation of HAAs in the liver and/or prostate is important for increased risk of prostate cancer. However, considering its opposition to past findings, future studies testing larger Japanese cohorts are warranted to replicate these findings, and further research is needed to understand how *CYP1A2* polymorphisms metabolically influence HAA carcinogens.

There are several limitations of our study. First, an inherent challenge in case control studies is to minimize the influence of selection and information bias (e.g., recall bias), although in our study, the controls were not only population- and age-matched but also were recruited from the same city area (metropolitan Tokyo). Second, although we analyzed each individual polymorphism, we did not investigate the putative effects of different polymorphism combinations because of the modest sample size of our cohorts. Third, because we did not directly determine HAA metabolic reactions in prostate tissue or other organs such as the liver, the effects or contributions of other metabolic pathways cannot be excluded. Nevertheless, to our knowledge, this is the first study to investigate the relationship between HAA intake, functional gene polymorphisms related to the HAA metabolic pathway, and prostate cancer risk in Japanese men.

## Conclusions

In summary, we performed a secondary data analysis of an observational case control study to determine the relationships between HAA intake and specific polymorphisms in three genes to prostate cancer in Japanese men. Based on our analysis, we found that high HAA intake was significantly associated with increased prostate cancer risk. In addition, we found that the *NAT2* slow acetylator phenotype, the *CYP1A1* GA + GG genotype, and the *CYP1A2* CA + AA genotype were associated with increased prostate cancer risk. In particular, the *CYP1A1* GA + GG genotype had increased risk even in the low and moderate HAA intake groups. These findings indicate that high HAA intake is a risk factor of prostate cancer in Japanese men, and that the genotypes of *NAT2*, *CYP1A1*, and *CYP1A2* may affect the degree of cancer risk via changes in HAA-metabolizing enzymes.

## Abbreviations

4,8-DiMelQx: 2-Amino-3, 4, 8-trimethylimidazo[4, 5-f]quinoxaline
7,8-DiMelQx: 2-Amino-3, 7, 8-trimethylimidazo[4, 5-f]quinoxaline
95% CI: 95% Confidence interval
BMI: Body mass index
CYP: Cytochrome P450
FFQ: Food frequency questionnaire
HAA: Heterocyclic aromatic amine
IQ: 2-Amino-3-methylimidazo[4, 5-f]quinoline
MeIQ: 2-Amino-3, 4-dimethylimidazo[4, 5-f]quinoline
MeIQx: 2-Amino-3, 8-dimethylimidazo[4, 5-f]quinoxaline
NaCl: Sodium chloride
*NAT1*: *N*-acetyltransferase 1
*NAT2*: *N*-acetyltransferase 2
OR: Odds ratio
PhIP: 2-Amino-1-methyl- 6-phenylimidazo[4,5-b]pyridine
SD: Standard deviation
SNP: Single nucleotide polymorphism
Trp-P-1: 3-Amino-1, 4-dimethyl-5H-pyrido[4, 3-b]indole
UTR: Untranslated region
