# Correction to: Association between *NAT2*, *CYP1A1*, and *CYP1A2* genotypes, heterocyclic aromatic amines, and prostate cancer risk: a case control study in Japan

**DOI:** 10.1186/s12199-018-0718-z

**Published:** 2018-07-04

**Authors:** Masahide Koda, Motoki Iwasaki, Yuko Yamano, Xi Lu, Takahiko Katoh

**Affiliations:** 1 Department of Public Health, Faculty of Life Sciences, Kumamoto University, 1-1-1 Honjou, Chuo-ku, Kumamoto, 860-8556 Japan; 2 Division of Epidemiology, Center for Public Health Sciences, National Cancer Center, Tokyo, Japan; 3 Department of Hygiene and Preventive Medicine, Showa University School of Medicine, Tokyo, Japan

## Correction

Following publication of the original article [1], the authors reported a correction in the units in the methods section under “Subjects”

Originally, the sentence was stated as:

(ii) exhibited no evidence of prostate cancer as determined by blood test (PSA < 4 mg/dL)

The correct sentence is:

(ii) exhibited no evidence of prostate cancer as determined by blood test (PSA < 4 ng/ml)
